# Relationship between patient experience and hospital readmission: system-level survey with deterministic data linkage method

**DOI:** 10.1186/s12874-022-01677-8

**Published:** 2022-07-21

**Authors:** Eliza Lai-Yi Wong, Chin-Man Poon, Annie Wai-Ling Cheung, Frank Youhua Chen, Eng-Kiong Yeoh

**Affiliations:** 1 Centre for Health Systems and Policy Research, JC School of Public Health and Primary Care, Faculty of Medicine, The Chinese University of Hong Kong, Hong Kong SAR, China; 2 Department of Management Sciences, City University of Hong Kong, Hong Kong SAR, China

**Keywords:** Patient satisfaction, Data linkage, Data matching, Patient readmission, Quality of health care

## Abstract

**Background:**

Linkage of public healthcare data provides powerful resources for studying from a comprehensive view of quality of care than information for a single administrative database. It is believed that positive patient experiences reflect good quality of health care and may reduce patient readmission. This study aimed to determine the relationship between patient experience and hospital readmission at a system level by linking anonymous experience survey data with de-identified longitudinal hospital administrative admissions data.

**Methods:**

Data were obtained by linking two datasets with anonymised individual-level records from seven largest-scale acute public hospitals over seven geographical clusters in Hong Kong. Selected records in the two datasets involving patient experience survey (PES) (2013 survey dataset) and healthcare utilization (admissions dataset) were used. Following data cleaning and standardization, a deterministic data linkage algorithm was used to identify pairs of records uniquely matched for a list of identifiers (10 selected variables) between two datasets. If patient’s record from the survey dataset matched with the hospitalization records in the admissions dataset, they were included in the subsequent analyses. Bivariate analyses and multivariable logistic regression models were performed to evaluate the associations between hospital readmission in the next calendar month and patient experience.

**Results:**

The overall matching rate was 62.1% (1746/2811) for PES participants aged 45 or above from the survey dataset. The average score for overall inpatient experience was 8.10 (SD = 1.53). There was no significant difference between matched patients and unmatched patients in terms of their score for the perception of overall quality of care received during hospitalization (*X*^2^ = 6.931, *p*-value = 0.14) and score for overall inpatient experience (*X*^2^ = 7.853, *p*-value = 0.25). In the multivariable model, readmission through the outpatient department (planned admission) in the next calendar month was significantly associated with a higher score given to the overall quality of care received (adjusted OR = 1.54, 95%CI = 1.09–2.17), while such association was absent for readmission through Accident and Emergency department (adjusted OR = 0.75, 95%CI = 0.50–1.12).

**Conclusions:**

This study demonstrated the feasibility of routine record linkage, with the limited intrusion of patients’ confidentiality, for evaluating health care quality. It also highlights the significant association between readmission through planned readmission and a higher score for overall quality of care received. A possible explanation might be the perceived better co-ordination between outpatient departments and inpatient service and the well-informed discharge plan given to this group of patients.

## Background

With an emphasis on patient self-reported outcomes, increasing attention has been focused on patient experience to evaluate the quality of healthcare system [1]. Patient experience and satisfaction have been started since the 1990s in different jurisdictions, including the US [2], UK [3], and Australia [4]. Subsequently, the patient experience survey was conducted in Hong Kong (HK), the first Asian jurisdiction to launch a benchmark patient experience and satisfaction in 2010 [5]. It was followed by Singapore [6] and China [7]. A number of studies concluded that patient experience scores were more predictive of hospital readmission than clinical performance measures such as health status of the patients [8–11]. It is believed that positive patient experiences reflect good health care quality. However, there are inconsistent results about the relationship between patient experience and unplanned readmission [9, 12, 13]. Most of these studies were conducted at the hospital level [9, 10] or disease-specific [8], which limits the role of performance indicators in the healthcare system. In addition, it may encounter a technical issue of management when applying such data. To reduce information bias and protect patient privacy, patient experience surveys are usually conducted anonymously and handled by a third party. Those data are not integrated into the central health record system to protect patient’s right and avoid the survey response influencing patient’s care [5, 14]. It therefore recreates fragmented databases and hurdles the evaluation of the overall quality of healthcare system. In HK, the access to personal identifiers is strictly protected by the Privacy Ordinance, which protects the privacy of individuals concerning personal data and provides for matters incidental thereto or connected therewith; therefore, the use of health data to evaluate healthcare system performance poses challenges. Linkage of public healthcare data enables healthcare practitioners, policymakers, and researchers to access complementary sources of information and obtain a more comprehensive view of a patient’s care than information for either database alone [15]. However, the potential of integrated analysis using data of patient experience from an anonymous survey and longitudinal administrative hospital data has not yet been fully explored.

This study attempted to determine the association between patient experience and hospital readmission at the system level by linking anonymous survey data with de-identified hospital administrative data. It is hypothesized that there would be more unplanned readmissions among patients in the next calendar month with poorer experience in inpatient care of their index admission, probably due to less person-centred care and informed discharge planning. An assessment linking both patient experience survey data with hospital admission records would be essential to determine their interrelationship.

## Methods

### Patient experience data and inpatient readmission data

Data were obtained by linking two datasets with anonymized individual-level records in the public healthcare sector in HK. One contained patient experience survey (PES) data from 3,566 patients (survey dataset), who were HK residents and were discharged from seven largest-scale acute public hospitals over seven geographical clusters between 18 October and 5 December 2013 [16]. This was a territory-wide cross-sectional telephone survey using a locally validated short-form PES instrument, the “Short-form of Hong Kong Inpatient Experience Questionnaire (SF-HKIEQ)” [17, 18]. Eligible respondents of PES were patients aged 18 or above with at least one night staying in any of the selected hospitals, excluding those admitted to psychiatric, obstetric, dentistry, hospice, infirmary, paediatric and intensive care wards. To increase the representative and avoid duplicated voices from the same patient, the subsequent admission of the same patients during the survey period were therefore removed before the sampling. The survey revealed the patient experience by applying two evaluative items – overall quality of care received and the overall patient experience from several aspects of hospital care, including the provision of care by hospital staff, provision of different components of care and treatment, provision of information on leaving the hospital and overall impression from the patient perspective [16]. Besides those evaluative questions for patient experience, the matched hospital episode data from the administrative sources contained variables which indicated the demographic characteristics and the related discharged information related to the respondents. These included (i) age, (ii) sex, (iii) residence district, (iv) admission source, (v) month of admission, (vi) month of discharge, (vii) admission specialty, (viii) discharge specialty, (ix) discharge hospital, and (x) length of stay (“List of Identifiers”). However, it didn’t link up to the any diagnosis or procedure details for the related discharged.

Another dataset (admissions dataset) was extracted from public hospital administrative admissions dataset, covering information of admission episodes among patients aged 45 and above in 2013 and 2014, which was originally used for another elderly study. They are patient-based records, and thus it also included the aforementioned List of Identifiers, the diagnosis code, and the procedure code of the admission episode. It also included an indicator for the respondents who received the government’s Comprehensive Social Security Assistant (CSSA). It refers to a financial assistance scheme for the local residents who receive supporting income up to a prescribed level to meet their basic needs. It is a common shadow reference of socioeconomic status among the local population. A pseudo identification number was available in this dataset to determine the admission episodes from the same patient.

### Data cleaning and preparation for linking datasets

Selected records in the two aforementioned datasets were used to prepare for record linkage. Out of 3566 respondents in the PES survey dataset, 2811 were aged 45 or above and included for record linkage. Those records from the survey dataset were treated as index admission in the linked database. In the admissions database with a total of 151,289 episodes among patients aged 45 or above, patients discharged home between October and December 2013 from the related hospitals were extracted for record linkage. Such data cleaning and preparation process would reduce the size of data management and the time required for record linkage. After being linked to the admissions dataset, those subsequent admission episodes in the next calendar month from the same patients would be identified as whether having readmission or not after the index admission.

**Fig. 1:**
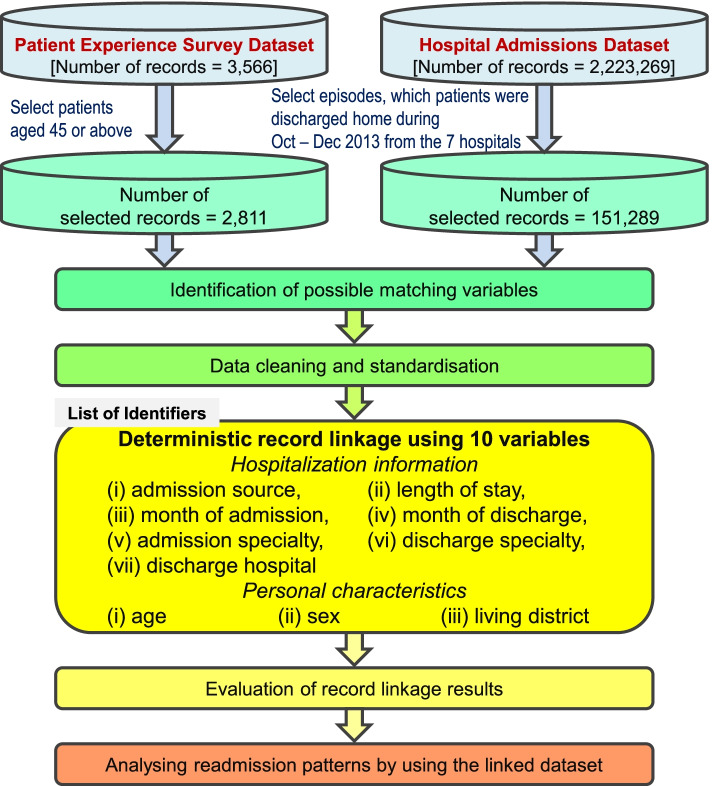
Flow chart of data selection and its linkage application

The primary outcome measure is whether the matched patients had an admission in the next calendar month after the index admission. The readmissions could be further categorized according to the admission sources, including those through Accident and Emergency department (A&E) and outpatient department. According to our healthcare system, the admission through A&E would be treated as unplanned readmission, while the admission through outpatient department referred as planned admission. The major covariates of interest included the responses from the participants who had completed the entire PES. The overall quality of care that the patient received was measured and was rated at five levels, namely “5: Excellent/Very Good”, “4: Good”, “3: Fair”, “2: Poor” and “1: Very poor”. An overarching question was included to enhance the sensitivity of response to the overall inpatient experience, which invited the patients to provide an overall experience score according to their summarized experience for their last hospital admission. The overall inpatient experience was measured with a score of 10 for the best experience and 0 for the worst experience. Since the median score of the overall inpatient experience was 8, the overall inpatient experience score was dichotomized into less than 8 and 8 or more in the analysis. Other covariates for the analyses were the demographic (included age, sex, whether receiving CSSA) and hospitalization information (included length of stay and discharge specialty) of index admission after the data matching. The discharge specialty was grouped into four categories: Medicine, Surgery, Oncology and Others. The discharges from Medicine, Surgery, and Oncology contributed to nearly 70% of the overall matched records. There was a variety of other discharge specialties, which included (i) cardiothoracic surgery, (ii) emergency medicine, (iii) ear, nose and throat, (iv) gynaecology, (v) neurosurgery, (vi) ophthalmology, (vii) orthopaedics and (viii) rehabilitation. However, the discharge proportion for each of those specialties were small, and they were thus grouped into “Others” for comparison in the study. Figure 1 summarizes the flow for data selection and its linkage application.

### Data linkage methods

Following data cleaning and standardization, a total of 10 matching variables in the List of Identifiers were identified for data linkage. The List of Identifiers in both the survey and admissions datasets did not contain any missing data. A deterministic data linkage algorithm was used to identify pairs of records uniquely matched for all ten variables between the survey and the admissions datasets. Patients with matched admission records were included in the following analyses (Relationship between Patient Experience and Hospital Readmission).

### Analysis of linkage accuracy and statistical analysis using linked data

First, we assessed the matching rate (1:1 matching) of PES survey dataset linked to admission dataset. Second, we evaluate data linkage quality by assessing the difference in score for overall quality of care received and overall inpatient experience between matched and unmatched patients by Pearson’s *X*^2^ tests, which may inform potential sources of bias caused during the linkage process [19]. Finally, we created a linked PES-readmission database, which allows analysis of the readmission episode after the PES rating.

### Statistical analysis

Descriptive statistics of the demographics and hospitalization of matched patients were tabulated. Bivariate analyses were performed to evaluate the associations between having an admission in the next calendar month and quality of care in terms of overall quality of care received during the hospitalization and overall inpatient experience of the related admission, presented in crude odds ratio (OR) with 95% confidence interval (CI). Given the nature of the problem and data used in this work, multivariable binary logistic regression models, controlling for all potential available confounding variables included gender, age, recipient of social allowance (CSSA), length of stay and discharge specialty of the index episode for conducting PES were employed to model the outcomes of a categorical dependent variable (whether having an admission in the next calendar month at 0 and 1 level; ‘0’ for not having an admission and ‘1’ for having at least one admission). Two continuous variables: namely age and length of study were converted to categorical variables for model fitting. For the length of stay, the cutoff value were referred to the average length of study in local public hospitals [20]. All linkage and statistical analyses were conducted in R version 3.5.2. The strength of evidence was considered strong against the null hypothesis, with a significant statistical difference when P values was less than 0.05 [21, 22].

## Results

### Accuracy of data linkage between PES and admission data

A total of 1746 patients who participated in the PES survey dataset had an admission episode recorded in the public hospital administrative admissions database, which were uniquely and perfectly matched on all ten variables. A total of 1,065 cases were unmatched (*n* = 69; 6.5%) or one to many (1:M) matched (*n* = 996; 93.5%). It gave an overall matching rate at 62.1% (1746/2811) for PES participants aged 45 or above.

### Demographics

Descriptive statistics about demographic characteristics, discharge episode and patient experience, and satisfaction for the matched patients are shown in Table 1. The median age of the matched patients were 65 years old (IQR = 56–76) and there were more males (52.6%). The median length of stay was 4 days, similar to the average 5.29 days for patients discharged from general hospitals in the early 2010s [23]. Most (661/1746; 37.9%) were discharged from Medicine specialty, followed by Surgery specialty (421/1746; 24.1%). The average score for overall experience was 8.10 (standard deviation = 1.53). To assess the potential bias caused, there was no significant difference between matched patients and unmatched patients in terms of their scores for the perception of overall quality of care received (*X*^2^ = 6.931, *p*-value = 0.14) and score for overall patient experience on inpatient service (*X*^2^ = 7.853, *p*-value = 0.25).

**Table 1:** Demographics, hospitalization information, overall quality of care received and overall inpatient experience of matched patients and their readmission patterns

		Patients having an admission episode in the next calendar month following the survey through…					
	Number of respondents	Any department		Readmission through A&E department		Readmission through referral from Outpatient department	
Variable		Number	(%)	Number	(%)	Number	(%)
**Gender**							
Male	919	230	(25.0)	88	(9.6)	159	(17.3)
Female	827	186	(22.5)	57	(6.9)	132	(16.0)
**Age**							
45–54	368	84	(22.8)	15	(4.1)	70	(19.0)
55–64	504	129	(25.6)	37	(7.3)	100	(19.8)
65–74	382	79	(20.7)	28	(7.3)	56	(14.7)
75+	492	124	(25.2)	65	(13.2)	65	(13.2)
**Length of stay**							
1–3 days	877	164	(18.7)	53	(6.0)	116	(13.2)
4–7 days	479	130	(27.1)	42	(8.8)	95	(19.8)
7 + day	390	122	(31.3)	50	(12.8)	80	(20.5)
**CSSA** ^a^							
No	1525	357	(23.4)	119	(7.8)	256	(16.8)
Yes	221	59	(26.7)	26	(11.8)	35	(15.8)
**Discharge specialty**							
Medicine^b^	661	174	(26.3)	74	(11.2)	113	(17.1)
Surgery	421	105	(24.9)	34	(8.1)	74	(17.6)
Oncology	85	55	(64.7)	8	(9.4)	47	(55.3)
Others^c^	579	82	(14.2)	29	(5.0)	57	(9.8)
**Overall quality of care received**							
“Excellent/Very Good” / “Good”	1353	331	(24.5)	107	(7.9)	243	(18.0)
“Fair” / “Poor” / “Very poor”	393	85	(21.6)	38	(9.7)	48	(12.2)
**Overall inpatient experience**							
Higher than or equal 8	1303	316	(24.3)	105	(8.1)	230	(17.7)
Lower than 8	443	100	(22.6)	40	(9.0)	61	(13.8)

^a^CSSA represents “Comprehensive Social Security Assistance”

^b^Medicine specialty includes cardiac care unit, geriatrics, infectious disease and general medicine

^c^Others include specialty in (i) cardiothoracic surgery, (ii) emergency medicine, (iii) ear, nose and throat, (iv) gynaecology, (v) neurosurgery, (vi) ophthalmology, (vii) orthopaedics and (viii) rehabilitation

### Relationship between patient experience and hospital readmission

There was 416 matched patients with admission in the next calendar month after an index admission for PES, giving a readmission rate at 23.8%. Separately, about three-quarters of patients (77.5%; 1353/1746) rated the overall quality of care received as “Excellent/Very Good” or “Good”, while a similar proportion (74.6%; 1303/1746) gave a score of 8 or above for the overall patient experience of the inpatient services received. There was no significant difference in the number of patients having readmission in the ensuing calendar month following PES as compared with those who gave a lower score for overall quality of care received (Crude OR = 1.17, 95%CI = 0.90–1.54) and overall inpatient experience (Crude OR = 1.09, 95%CI = 0.85–1.42). Bivariate analyses found that longer length of stay (*X*^2^ = 27.5, *p*-value < 0.01) and discharge from medical, surgical and oncology wards were associated with readmission (Crude OR = 2.43, 95%CI = 1.86–3.17), as compared with those discharged from other specialties. Also, the age group of 65–74 and 75 or above, and discharge specialty from medicine, oncology and surgery were found to be significantly associated with readmission through the outpatient department, which was planned admission.

In the multivariable model controlling for all potential available confounding factors in term of age, length of stay and discharge specialty (Table 2), readmission through the outpatient department (planned admission) in the next calendar month was significantly associated with a higher score given to the overall quality of care received (adjusted OR = 1.54, 95%CI = 1.09–2.17), while such association was absent for readmission through A&E department (adjusted OR = 0.75, 95%CI = 0.50–1.12). A higher score given to the overall patient experience on inpatient service was less likely to have readmission through A&E department (adjusted OR = 0.86, 95%CI = 0.58–1.27) and more likely outpatient department (adjusted OR = 1.27, 95%CI = 0.93–1.75), but the results were not statistically significant (Table 3).

**Table 2:** Multivariate analyses on association of the readmission through different departments and overall quality of care received of index admission

	Patients having a readmission in the next calendar month through		
	**Any department**	**Readmission through A&E department**	**Readmission through referral from Outpatient department**
	**Adjusted OR (95%CI)**	**Adjusted OR (95%CI)**	**Adjusted OR (95%CI)**
**Intercept**	0.27 (0.16–0.46)*	0.09 (0.04–0.21)*	0.15 (0.08–0.28)*
**Gender**			
Male	Ref	Ref	Ref
Female	0.94 (0.75–1.19)	0.71 (0.49–1.01)	1.01 (0.77–1.32)
**Age**			
45–54	Ref	Ref	Ref
55–64	1.06 (0.76–1.49)	1.74 (0.93–3.23)	0.96 (0.67–1.37)
65–74	0.8 (0.55–1.15)	1.63 (0.85–3.14)	0.66 (0.44–0.99)*
75+	1.1 (0.78–1.54)	3.25 (1.81–5.86)	0.62 (0.42–0.91)*
**Length of stay**			
1–3 days	Ref	Ref	Ref
4–7 days	1.41 (1.07–1.86)*	1.25 (0.81–1.92)	1.47 (1.08–2.02)*
8 + days	2.02 (1.52–2.69)*	2.02 (1.33–3.06)*	1.81 (1.3–2.52)*
**CSSA** ^**a**^			
No	Ref	Ref	Ref
Yes	1.11 (0.79–1.55)	1.38 (0.87–2.19)	0.89 (0.59–1.34)
**Discharge specialty**			
Others^b^	Ref	Ref	Ref
Medicine^c^	2.08 (1.56–2.79)*	1.82 (1.18–2.81)*	2.11 (1.5–2.99)*
Oncology	11.18 (6.73–18.56)*	2.07 (0.91–4.72)	11.08 (6.62–18.54)*
Surgery	1.94 (1.41–2.67)*	1.34 (0.82–2.21)	2.08 (1.43–3.02)*
**Overall quality of care received**			
“Fair” / “Poor” / “Very poor”	Ref	Ref	Ref
“Excellent/Very Good” / “Good”	1.12 (0.84–1.48)	0.75 (0.5–1.12)	1.54 (1.09–2.17)*

* *P* < .05

^a^CSSA represents “Comprehensive Social Security Assistance”

^b^Others include specialty in (i) cardiothoracic surgery, (ii) emergency medicine, (iii) ear, nose and throat, (iv) gynaecology, (v) neurosurgery, (vi) ophthalmology, (vii) orthopaedics and (viii) rehabilitation

^c^Medicine specialty includes cardiac care unit, geriatrics, infectious disease and general medicine

**Table 3:** Multivariate analyses on association of the readmission through different departments and overall inpatient experience of index admission

Patients having a readmission in the next calendar month through			
	**Any department**	**Readmission through A&E department**	**Readmission through referral from Outpatient department**
	**Adjusted OR (95%CI)**	**Adjusted OR (95%CI)**	**Adjusted OR (95%CI)**
**Intercept**	0.29 (0.17–0.48)*	0.08 (0.03–0.18)*	0.18 (0.1–0.33)*
**Gender**			
Male	Ref	Ref	Ref
Female	0.93 (0.74–1.18)	0.72 (0.5–1.02)	0.99 (0.76–1.29)
**Age**			
45–54	Ref	Ref	Ref
55–64	1.06 (0.76–1.48)	1.76 (0.94–3.27)	0.94 (0.65–1.35)
65–74	0.8 (0.55–1.15)	1.65 (0.86–3.17)	0.66 (0.44–0.99)*
75+	1.1 (0.79–1.54)	3.24 (1.8–5.84)*	0.62 (0.42–0.91)*
**Length of stay**			
1–3 days	Ref	Ref	Ref
4–7 days	1.41 (1.07–1.86)*	1.26 (0.82–1.94)	1.46 (1.07–2)*
8 + days	2.02 (1.52–2.69)*	2.01 (1.33–3.06)*	1.82 (1.31–2.54)*
**CSSA** ^a^			
No	Ref	Ref	Ref
Yes	1.1 (0.79–1.54)	1.39 (0.87–2.2)	0.88 (0.59–1.32)
**Discharge specialty**			
Others^b^	Ref	Ref	Ref
Medicine^c^	2.08 (1.55–2.78)*	1.82 (1.18–2.82)*	2.09 (1.48–2.96)*
Oncology	11.22 (6.76–18.63)*	2.04 (0.9–4.65)	11.14 (6.66–18.64)*
Surgery	1.94 (1.41–2.67)*	1.34 (0.81–2.2)	2.08 (1.43–3.02)*
**Overall inpatient experience**			
<8	Ref	Ref	Ref
>=8	1.04 (0.8–1.36)	0.86 (0.58–1.27)	1.27 (0.93–1.75)

* *P* < .05

^a^CSSA represents “Comprehensive Social Security Assistance”

^b^Others include specialty in (i) cardiothoracic surgery, (ii) emergency medicine, (iii) ear, nose and throat, (iv) gynaecology, (v) neurosurgery, (vi) ophthalmology, (vii) orthopaedics and (viii) rehabilitation

^c^Medicine specialty includes cardiac care unit, geriatrics, infectious disease and general medicine

## Discussion

### Principal results

We have established a large dataset linking patient experience and hospital readmission data. The study would try to apply data linkage of patient experience data indicating the improvement actions for the patient care. A matching rate of 62% was achieved through the deterministic data linkage method. There was no significant difference in the score for perception of overall quality of care received and the score for overall patient experience on inpatient service between matched dataset and un-match dataset. To the best of our knowledge, this is the first study to adopt the deterministic data linkage method to examine the relationship of patient experience with hospital readmission in the context of Chinese population. The findings indicated that a few patient’s characteristics were associated with better quality of care.

### Data linkage applications

Administrative data of health care organization provides an important resource for healthcare system performance evaluation. It involves longitudinal data from the patient’s first contact point of the healthcare system, thus it provides easy-to-access data for follow-up analysis and trend comparison. However, the datasets, which are usually organized in different entities and different premises for administrative purpose, pose data integration and analysis challenges. In addition, it would not involve the evaluation information from a patient perspective. Thus, data linkage between patient experience from the patients and hospital admission would be an important indicator of quality of care. Despite the importance of linking big data, the linkage of public health data is limited in different jurisdictions, including HK, because the privacy ordinance severely regulates access to personal identifiers. Therefore, instead of using unique personal identifiers, we tried to assess the linkage between the patient experience survey data and hospital admissions data through the deterministic data linkage method. It provided input research for the longitudinal, valid and reliable mechanism to monitor patient’s care over time with the confidentiality requirement [24–27]. It may highlight the possible inadequate governance of the accuracy of data and lack of standardization of completeness of the data within each data source. For many variables examined in the reported studies, the evidence for an association between the variable and rates of data linkage was inconsistent, probably due to different sampled populations and other contextual factors. Thus, further studies are needed to review its application with better quality of data in future.

### Correlation between patient experience and hospital readmission

 This study found that patients with better quality of care received had 1.5 times more for planned hospital readmission. Planned hospital readmission was defined as follow-up for ambulatory care, especially for medicine, oncology and surgery disciplines. With this support, those patients with higher scores of overall inpatient experience were less likely to have emergency-associated hospital readmission. It may suggest the higher score inpatient experience with responsive care during hospitalization for better post-discharge self-care management behind the associations between patient experience and emergency admission. It aligned with other studies that better patient experience with responsive care may influence hospitalization rates [11]. Thus, the staff’s responsiveness was important, particularly for those older age and with chronic diseases together with possible complications, especially those discharged from medicine, oncology and surgery discipline. Staff responsiveness maybe an additional tool to assess the hospital performance in term of post-discharge and readmission [11]. Also, other hospital-level studies have found that high hospital readmission rates were associated with lower patient experience scores in a mixed surgical patient population including pain control and colorectal surgery population [28]; and lower experience scores with discharge preparedness in vascular surgery patients [8].

### Limitations

This study carries a few limitations. First, selection bias could not be ruled out as this study evaluated the readmission patterns among a subset of respondents from the patient experience survey, who could be matched after data linkage. The applied patient experience survey had a slight tendency in favour of the younger age groups and those living in the community [16]. Second, there was limited information in either dataset to determine whether the readmission was avoidable. Third, the interpretation of the readmission results in this study was also limited by the level of details in the database, which only allow us to define a readmission by having hospitalization episodes in consecutive calendar month instead of the more generalised use of 28 days or 30 days in the context of measurement of hospital readmissions. Also, the list of factors controlled in the model was limited by the availability and comprehensiveness of data. This limitation is also reflected by the low values of R squared for the models in Tables 2 and 3, which are in the range between 0.051 and 0.072. Fourthly, due to the limited details in the hospital report, we could not perform further analyses on the correlation between each measured item and readmission rates. However, we performed subgroup analysis in term of demographics and medical condition; and the patient perception of quality of care were adjusted for variations in age, length of stay and discharge speciality.

## Conclusions

This study highlights the significant association between readmission through outpatient readmission and a higher score for perceived quality of care. A possible explanation might be the perceived better coordination between outpatient departments and inpatient service and the well-informed discharge plan given to this group of patients. Therefore, patient-reported experience of hospital performance can have an important role in evaluating and improving the healthcare system. Methodologically, this study demonstrated the feasibility of routine record linkage, with the limited intrusion of patients’ confidentiality, for evaluation of the health care quality.

## Data Availability

The datasets generated and/or analysed during the current study are not publicly available. They contain information that could compromise research participant privacy but are available from the corresponding author on reasonable request.
